# CEO Overconfidence and Corporate Innovation Outcomes: Evidence From China

**DOI:** 10.3389/fpsyg.2022.760102

**Published:** 2022-02-07

**Authors:** Zhongze Li, Yi Zhang

**Affiliations:** ^1^School of Accounting, Nanjing Audit University, Nanjing, China; ^2^School of Management, Nanjing University, Nanjing, China

**Keywords:** CEO overconfidence, innovation outcomes, Chinese context, ownership structure, CEO attributes

## Abstract

This study examines how chief executive officer (CEO) overconfidence can influence the quantity, quality and direction of corporate innovation using Chinese firms for the period 2009–2016. Our results suggest that overall, CEO overconfidence has a positive impact on firm innovation productivity. Furthermore, this effect is significant for Chinese non-SOEs but not for Chinese SOEs. Specifically, an overconfident CEO can facilitate firm innovation in new technological areas but not in the firm’s existing areas. Additionally, we find that internal controls can regulate the relationship between CEO overconfidence and innovation. Interestingly, when the internal control level is too high or too low, the correlation between CEO overconfidence and innovation productivity is not significant.

## Introduction

It is well recognized that innovation is vital for a firm’s growth and long-term competitive advantage (Porter, 1992; Loukil et al., 2020). Chief Executive Officer (CEO) overconfidence is considered to be highly related to firm innovation since overconfident CEOs are likely to take risks, address challenges, and implement corporate changes such as investing in R&D activities (Malmendier and Tate, 2005a,b; Galasso and Simcoe, 2011; Hirshleifer et al., 2012; Nowak, 2018). Despite the fact that CEO overconfidence has been extensively discussed, especially with regard to its effects on firm innovation, most existing studies have analyzed the influence of CEO overconfidence on firm innovation in the Western context. Francis and Smith (1995) find that CEOs in developed markets tend to better adopt changes and to implement risky projects than those in emerging markets. Additionally, Gelfand et al. (2007) argue that every organization is inevitably influenced by the particular national culture in which it is located. Therefore, previous findings on CEO overconfidence and firm innovation are not necessarily applicable to the Chinese context. In fact, there is no empirical study of this question based on the Chinese context.

As important firm activities, innovation activities could be heavily influenced by CEOs’ characteristics (e.g., Lu and Wang, 2017). Among various CEO characteristics, CEO overconfidence is considered to be highly related to firm innovation since overconfident CEOs are likely to take risks, address challenges, and implement corporate changes such as investing in R&D activities (e.g., Galasso and Simcoe, 2011; Hirshleifer et al., 2012). In light of these arguments, we expect a positive association between CEO overconfidence and innovation.

Our paper contributes to the literature in several dimensions. First, we add to the literature on the determinants of corporate innovation based on the Chinese context (for a comprehensive review, see He and Tian (2018)). Our study adds to the literature by considering firm ownership heterogeneity, namely, SOEs and non-SOEs, as an important institutional factor in the innovation-generating process. Second, prior studies on CEO overconfidence and firm innovation focus mainly on innovation efforts and innovation productivity (Malmendier and Tate, 2005a,b; Galasso and Simcoe, 2011; Hirshleifer et al., 2012). In our study, we additionally test the effects of CEO overconfidence on the quality and direction of innovation. Our research findings on CEO overconfidence and innovation quality and direction have potential implications for human resource management. For instance, a fast-growing high-tech firm could hire a more overconfident CEO since an overconfident CEO, on average, will spur innovation in new areas. Finally, to the best of our knowledge, we provide the first findings on the effects of CEO overconfidence on innovation considering firms’ internal control quality.

The rest of this article is organized as follows. In section “Hypothesis Development,” we discuss the hypothesis development. In section “Date and Research Methodology,” we introduce the data and methods; in section “Empirical Results,” we discuss the empirical results; and in section “Robustness Tests,” we conduct robustness tests. In the last section, we conclude the study.

## Hypothesis Development

In recent years, corporate innovation has become an increasingly important research topic that has attracted a great deal of attention and academic research effort from researchers in various disciplines (e.g., Crossan and Apaydin, 2010; Honore et al., 2015; Huo et al., 2017; He and Tian, 2018; for a comprehensive review, see He and Tian (2018)). Prior studies show that CEOs are the most important decision makers in firms, especially public firms (He and Tian, 2018; Liu et al., 2018). As a result, CEOs’ backgrounds, leadership styles and personal attributes might have substantial impacts on firm performance and outcomes (Waldman et al., 2001; Hambrick, 2007; Ling et al., 2008). As one of the most important activities for firms, corporate innovation could be heavily influenced by CEOs’ personal characteristics (Nadkarni and Herrmann, 2010; Lu and Wang, 2017; Sunder et al., 2017; Zhao and Xie, 2020). For instance, Sunder et al. (2017) find that firms run by CEOs with pilot experience generate more patents and citations, which indicates higher innovation efficiency.

Upper echelons theory supports that management attributes, such as CEOs’ attributes, could influence corporate decisions and organizational performance outcomes (Hambrick and Mason, 1984; Hambrick, 2007). According to this theory, CEOs are considered to be boundedly rational. Their personalities and values are relatively permanent, and their decisions are inevitably influenced by their personal attributes (Finkelstein et al., 2009). Several studies based on upper echelons theory find evidence that CEOs’ background features, personality characteristics and leadership styles have a significant effect on corporate strategic dynamism (Crossland et al., 2014); R&D spending (Barker and Mueller, 2002); mergers and acquisitions (Yim, 2013); corporate risk-taking propensity (Serfling, 2014); and firm profitability (Wang et al., 2016).

As important firm activities, innovation activities could be heavily influenced by CEOs’ characteristics (Nadkarni and Herrmann, 2010; Lu and Wang, 2017; Sunder et al., 2017). For instance, Sunder et al. (2017) find that firms run by CEOs with pilot experience generate more patents and citations, which indicates higher innovation efficiency, than those run by CEOs without pilot experience.

Among various CEO characteristics, CEO overconfidence is considered to be highly related to firm innovation since overconfident CEOs are likely to take risks, address challenges, and implement corporate changes such as investing in R&D activities (Malmendier and Tate, 2005a,b; Galasso and Simcoe, 2011; Hirshleifer et al., 2012; Nowak, 2018). Firstly, overconfident CEOs tend to have optimistic estimates about the company’s operating conditions, so they will tend to ignore the company’s current risks and use more resources for the company’s long-term development (Nowak, 2018). Enterprise innovation investment belongs to part of the company’s long-term development investment, so it will also get corresponding investment and finally enjoy growth; Furthermore, overconfident CEOs prefer to face challenges and take risks (Malmendier and Tate, 2005a,b). There are high risks and uncertainties in enterprise R&D activities (Wyatt, 2005), so they can arouse and meet the challenge desire of overconfident CEOs. Overconfident CEOs often obtain professional satisfaction from high-risk activities such as enterprise R&D (Hirshleifer et al., 2012). Finally, overconfident CEOs tend to be more optimistic when making enterprise decisions, and will consider the company’s operation in the longer term (Galasso and Simcoe, 2011). This long-term decision-making thinking can also promote R&D investment and enterprise innovation to a certain extent.

In light of these arguments, we expect a positive association between CEO overconfidence and innovation. Therefore, we develop the following hypothesis:

Hypothesis 1: Ceteris paribus, CEO overconfidence is positively associated with firm innovation.

Every firm is heavily influenced by a particular national institution in which it is located (Gelfand et al., 2007). The unique institutional background of China may affect the validity of applying Western-based empirical research findings on enterprise innovation to the Chinese context (Kim et al., 2010). In China, SOEs play a very important role in supporting the state’s policies (Wei, 2021). Many Chinese firms are SOEs and are controlled by SASAC (Bai et al., 2006; Bruton et al., 2015; The State-owned Assets Supervision and Administration Commission of the State Council [SASAC], 2018). In addition, in many Chinese SOEs, CEOs *de facto* government officials (Wei, 2021). Therefore, the Chinese government has a powerful influence on SOEs’ decision-making process. In this way, when a CEO of a Chinese SOE makes decisions on the firm’s management and operations, he/she would consider the government and SASAC’s ideas rather than his/her own. Therefore, the influence of CEOs’ personal characteristics, for example, overconfidence, on the companies’ operation, and management decision-making process are significantly weakened and constrained.

On the one hand, SASAC appoints SOE CEOs. The goal of SASAC is not to maximize economic benefits but to achieve political goals. The behavior of these CEOs is subject to the direction of SASAC. On the other hand, China’s state-owned assets supervision system is unique (SASAC, Commission for Discipline Inspection, audit office, etc.). CEOs are supervised by all the parties mentioned above, and enterprise innovation is an activity with high investment, high risk and a long return cycle, which also restrains the impact of CEO overconfidence on firm innovation.

In contrast to their state-owned counterparts who are directly controlled and heavily influenced by SASAC, Chinese private enterprises are more independently operated. CEOs of Chinese private enterprises are in charge of the firms’ daily operations and have a more powerful influence on the firm’s decision-making process than CEOs of Chinese SOEs. Generally, a CEO of a Chinese private enterprise can make decisions more independently than that of an SOE. Therefore, CEOs’ personal characteristics are less constrained and are more influential on the firm’s decision-making process.

In light of these arguments, we expect no association between CEO overconfidence and innovation in Chinese SOEs; in contrast, we expect a positive association between CEO overconfidence and innovation in Chinese private enterprises. Therefore, we develop the following hypothesis:

Hypothesis 2a: Ceteris paribus, in Chinese SOEs, CEO overconfidence is not associated with firm innovation.

Hypothesis 2b: Ceteris paribus, in Chinese private enterprises, overconfidence is positively associated with firm innovation.

## Date and Research Methodology

### Sample Selection and Sources of Data

The empirical analysis is performed on firms listed on the Shanghai and Shenzhen stock exchanges in China between 2009 and 2016. CEO characteristics, corporate governance data, firm-level financial statement data and trading data are collected and merged from the China Stock Market and Accounting Research database (CSMAR) and Wind Economic Database (WIND). Patent data are manually collected from the Chinese Patent Database (CNPAT) and double-checked to ensure accuracy. We first eliminate observations with missing values and then exclude Chinese special treatment firms (ST firms) and financial firms. After winsorizing all continuous variables at their 1st and 99th percentiles, we obtain a final sample of 6,327 observations.

### Dependent Variables

Our dependent variable is innovation productivity, LN_CITATIONS. Prior empirical research on innovation has heavily relied on patent data, especially for patent accounts and citations (e.g., He and Tian, 2013, 2018). Benjamin et al. (2016) argue that patents with citations are usually more qualified than those without citations. Therefore, we use the total number of patents granted that were cited at least once during a year and exclude patents without any citations during a year as an overall proxy for innovation productivity.

In our additional analysis, patents are classified into patents with citation rates in the top 1%, those in the top 2–10%, those below the top 10% but with at least one citation, and those with no citations. We believe that the higher a patent’s citation rate is, the higher its economic value and therefore the higher its quality. Meanwhile, according to whether the patents applied for by firms belong to fields familiar to the firm, this paper divides the patents applied for by firms into breakthrough innovation or conservative innovation. It is believed that breakthrough innovation can bring more development opportunities to enterprises than conservative innovation, so the quality of breakthrough innovation is also higher.

### Independent Variables

According to Hoffrage (2004), overconfidence is defined as the phenomenon that people’s confidence in their judgments and knowledge is higher than the accuracy of these judgments. Similarly, CEO overconfidence refers to the phenomenon that a CEO’s confidence in their judgments and knowledge is higher than the accuracy of his/her judgments. Prior studies reveal that CEO overconfidence is a personality characteristic that is heavily influenced by the CEO’s individual background, such as age, gender, formal education level, professional business education background and position duality. Older managers are more risk averse than younger ones, and they are more likely to understand their ability objectively (Hitt and Tyler, 1991; Yim, 2013; Serfling, 2014). Male managers are often more conceited and radical in business management than female managers (Byrnes et al., 1999). People with higher education levels are more confident in their ability and accuracy of judgment and more likely to show overconfidence than those with lower education levels. Additionally, a CEO’s formal education level may build his/her confidence and openness to ideas (Schrand and Zechman, 2011; Liu et al., 2018). Managers with professional business education backgrounds may have a deeper understanding of risks and benefits and be more cautious in making management decisions than those without such backgrounds. Therefore, a CEO with a professional economic or management education background tends to be less overconfident than a CEO without such a background (Malmendier and Tate, 2005a,b). For position duality, if a CEO is granted the position of chairman, it will potentially improve the CEO’s recognition of his own ability and further promote his overconfidence in decision-making (Schrand and Zechman, 2011).

Therefore, to operationally measure CEO overconfidence, we set the age indicator, gender indicator, formal education level indicator, professional business education background indicator and position duality indicator. If the CEO’s age is less than the average for the sample, the age indicator is assigned a value of 1, and 0 otherwise; if the CEO is male, the gender indicator is assigned a value of 1, and 0 otherwise; if the CEO has a bachelor’s degree or above, the educational indicator is assigned a value of 1, and 0 otherwise; if the CEO has professional management, business, or corporate law education background, the professional business education background indicator is assigned a value of 1, and 0 otherwise; and if the CEO is also chairman of the firm, the position indicator is assigned a value of 1, and 0 otherwise. We set up a dummy variable (OVER_CONFIDENCE) that equals 1 when a CEO is considered to be overconfident, that is, when the sum of the five indicators for the CEO is 4 or 5; otherwise, OVER_CONFIDENCE is 0.

### Control Variables

Based on prior literature, we control for firm size, financial characteristics, corporate governance characteristics, stock market trading characteristics, nature of ownership, and other characteristics of the enterprise. We add control variables to capture firm size, performance effects and other financial characteristics, such as asset size (SIZE), the asset liability ratio (LEV), capital intensity (INTEN), fixed assets (FIXED_ASSET), operating income before depreciation (ROA), net operating cash flow (CASH_FLOW), retained earnings ratio (RE_EARNING), the growth rate of operating income (GROWTH), annual earnings per share (RET), the annual volatility of the stock price (VOLATILITY), ownership concentration (OWNCON), and the executive shareholding ratio (EXCHH) (Frost, 1997; Healy and Palepu, 2001; Ben-Amar and Zeghal, 2010; Lee et al., 2016; Loukil et al., 2020; Huang and Yuan, 2021). In addition, we add other control variables, such as the percentage of independent directors (INDEP) (Jiraporn et al., 2018) and the number of board meetings held each year (MEETING), to capture the firm’s internal control level. Additionally, considering the differences between state-owned enterprises and private enterprises in China, we add a dummy variable SOE; if the firm is state-owned, the dummy variable equals 1, and 0 otherwise. Detailed definitions of the control variables are listed in Table 1.

**TABLE 1:** Variable definitions.

Variable	Definition
**Dependent variables**	
LN_CITATIONS	Natural log of the number of patents eventually granted with citations
LN_TOP1	Natural log of the number of patents with a citation rate in the top 1%
LN_TOP2_10	Natural log of the number of patents with a citation rate in the top 2–10%
LN_CITED	Natural log of the number of patents with at least one citation and a citation rate lower than the top 10%
LN_UNCITED	Natural log of the number of patents without citations
LN_NEWCLASS	Natural log of the number of patents in new areas
LN_KNOWNCLASS	Natural log of the number of patents in familiar areas
**Independent variables**	
OVER_CONFIDENCE	See the definition in 3.3 Independent variables
**Control variables**	
SIZE	Natural log of the book value of total assets
LEV	Natural log of total debts scaled by total assets
INTEN	Natural log of revenue scaled by total assets
ROA	Natural log of net income scaled by total assets
FIXED_ASSET	Property, plant and equipment, scaled by total assets
RE_EARNING	Natural log of the retained earnings ratio: the proportion of the sum of surplus reserves and undistributed profits to total assets
GROWTH	The annual revenue growth rate
CASH_FLOW	Net cash flow from operating activities, scaled by the firm’s market value
RET	Nature log of annual return on equity considering the reinvestment of cash dividends
VOLATILITY	Annual volatility of share price: Variance in the monthly return rate of stocks in the current year
OWNCON	Natural log of equity concentration: proportion of the top 10 shareholders
EXCHH	Natural log of the proportion of shares held by senior management: Number of shares held by senior management/number of shares in circulation
INDEP	Natural log of the proportion of independent directors: number of independent directors/number of board of directors
MEETING	Natural log of the number of board meetings held each year
SOE	State-owned firm: 1; otherwise: 0

### Model Specification

To test the impact of CEO overconfidence on the innovation of listed firms in China, this paper constructs the following measurement model:


(1) $$LN\_CITATIONS=\alpha +\beta OVER\_CONFIDENCE_{i,t-1}+\gamma X_{i,t-1}+\lambda_{i}+\lambda_{t-1}+\mu_{i,t-1}$$

where LN_CITATIONS is the measure of innovation productivity: the logarithm of the number of patents eventually granted with citations for firm i in year t-1. Prior studies (Tang and Peng, 2003; Chen, 2013) have introduced a lag structure into the models to account for the time lag between CEO decisions and proxies for firm innovation. Following Balsmeier et al. (2014) and Loukil et al. (2020), we consider a 1-year lag since it takes time for CEO decisions to change firm outcomes. Accordingly, the time period for the independent variables and the control variables is 2009–2015, while the time period for the dependent variables is 2010–2016. To avoid the omission of important explanatory variables and endogeneity problems caused by reverse cause and effect, the measurement model in this paper includes financial indicators, corporate governance indicators, ownership nature and other indicators as control variables. Industry fixed effects (λi) and year fixed effects (λt) are added to the model.

### Descriptive Statistics and Correlation Matrix

Table 2 presents the descriptive statistics for the key variables in this paper. From the statistical results, we can see that the mean and the standard deviation of LN_CITATIONS are 1.400 and 1.892, respectively, with values ranging from 0 to 8.783. The standard deviation is 1.892, which indicates that there are significant differences in the productivity of corporate innovation among listed firms in China. The mean of OVER_CONFIDENCE is 0.474, indicating that the proportion of overconfident CEOs among CEOs of listed companies in China is 47.4%.

**TABLE 2:** Descriptive statistics.

VarName	Obs	Mean	*SD*	Min	P25	Median	P75	Max
LN_CITATIONS	6,327	1.400	1.892	0.000	0.000	0.000	3.045	8.783
OVER_CONFIDENCE	6,327	0.474	0.499	0.000	0.000	0.000	1.000	1.000
SIZE	6,327	21.942	1.087	19.981	21.149	21.793	22.549	25.949
LEV	6,327	0.409	0.192	0.047	0.253	0.402	0.557	0.845
INTEN	6,327	2.091	1.264	0.397	1.242	1.765	2.575	8.840
ROA	6,327	0.057	0.042	–0.061	0.026	0.051	0.081	0.219
FIXEDASSET	6,327	0.225	0.149	0.003	0.108	0.199	0.314	0.698
GROWTH	6,327	25.419	24.458	–0.455	9.086	18.454	33.732	170.380
CASH_FLOW	6,327	0.021	0.031	–0.081	0.004	0.018	0.038	0.123
RE_EARNING	6,327	0.181	0.102	–0.191	0.113	0.169	0.239	0.546
RET	6,327	0.262	0.590	–0.533	–0.172	0.113	0.535	2.780
VOLATILITY	6,327	0.137	0.054	0.056	0.100	0.125	0.159	0.389
OWNCON	6,327	0.589	0.139	0.233	0.490	0.600	0.699	0.899
EXCHH	6,327	0.160	0.321	0.000	0.000	0.004	0.150	1.766
INDEP	6,327	0.370	0.050	0.333	0.333	0.333	0.400	0.571
MEETING	6,327	9.647	3.523	4.000	7.000	9.000	12.000	24.000

Table 3 presents the Pearson correlation coefficients for the main variables used in our regression analysis. The correlation between innovation (LN_CITATIONS) and CEO overconfidence (OVER_CONFIDENCE) is significantly positive, which is consistent with the argument that overconfident CEOs courage firm innovation. As expected, the innovation measure is also significantly correlated with most of the control variables.

**TABLE 3:** Correlation matrix.

	LN_CITATIONS	OVER_CONFIDENCE	SIZE	LEV	INTEN	ROA	FIXED_ASSET	GROWTH	CASH_FLOW	RE_EARNING	RET	VOLATILITY	OWNCON	EXCHH	INDEP	MEETING	SOE
LN_CITATIONS	1																
OVER_CONFIDENCE	0.069***	1															
SIZE	–0.018	−0.105***	1														
LEV	−0.033**	−0.104***	0.578***	1													
INTEN	−0.168***	0.040***	−0.054***	−0.205***	1												
ROA	0.129***	0.033**	−0.099***	−0.431***	−0.136***	1											
FIXED_ASSET	0.023	−0.070***	0.024	0.087***	−0.112***	−0.162***	1										
GROWTH	0.064***	0.059***	−0.028*	0.014	0.012	0.178***	−0.196***	1									
CASH_FLOW	0.027*	−0.081***	0.202***	0.055***	−0.146***	0.180***	0.335***	−0.176***	1								
RE_EARNING	0.074***	0.034**	−0.147***	−0.533***	−0.102***	0.680***	−0.024*	−0.100***	0.199***	1							
RET	−0.196***	0.059***	−0.073***	–0.010	0.056***	0.103***	−0.062***	0.048***	−0.054***	0.036**	1						
VOLATILITY	−0.191***	0.068***	−0.138***	−0.056***	0.134***	−0.056***	−0.127***	0.069***	−0.202***	−0.074***	0.481***	1					
OWNCON	0.059***	–0.021	–0.010	−0.164***	0.014	0.231***	−0.049***	0.063***	0.040***	0.138***	−0.053***	−0.049***	1				
EXCHH	0.049***	0.164***	−0.339***	−0.370***	0.090***	0.196***	−0.196***	0.131***	−0.178***	0.179***	0.085***	0.154***	0.096***	1			
INDEP	−0.026*	0.011	–0.000	−0.032**	0.054***	–0.012	−0.073***	0.036**	−0.047***	–0.016	0.010	0.033**	0.078***	0.101***	1		
MEETING	−0.082***	0.017	0.192***	0.182***	0.103***	−0.050***	−0.159***	0.145***	−0.158***	−0.174***	0.030**	0.130***	0.017	0.083***	0.052***	1	
SOE	0.008	−0.099***	0.313***	0.289***	−0.084***	−0.129***	0.143***	−0.067***	0.152***	−0.127***	−0.064***	−0.118***	−0.082***	−0.452***	−0.060***	−0.055***	1

*, **, and *** denote significance at the 10, 5, and 1% levels, respectively. Standard errors are corrected for heteroskedasticity and clustering at the CEO-firm level (t-statistics are in parentheses).

## Empirical Results

### Baseline Results

#### CEO Overconfidence and Firm Innovation Productivity

This paper adopts a step-by-step regression approach, focusing on the impact of CEO overconfidence on the productivity of corporate innovation. The first column of Table 4 shows that the correlation coefficient between CEO overconfidence and innovation productivity is 0.12, which is significantly positive at the 10% level. The control variables for corporate financial characteristics and corporate governance variables were gradually introduced, and the second and third columns of Table 4 show that the correlation coefficient between CEO overconfidence and innovation productivity is 0.17, which is significantly positive at the 1% level. This indicates that with the improvement of CEO overconfidence, the number of granted patents with citations will increase, which indicates that the CEO overconfidence attribute can improve the innovation productivity of enterprises to a certain extent; this result supports hypothesis H1.

**TABLE 4:** Baseline regression results.

VARIABLES	(1)	(2)	(3)
	LN_CITATIONS t + 1	LN_CITATIONS t + 1	LN_CITATIONS t + 1
OVER_CONFIDENCE	0.12*	0.17***	0.17***
	(1.853)	(2.810)	(2.778)
SIZE		0.32***	0.32***
		(7.790)	(7.146)
LEV		–0.65**	–0.32
		(–2.002)	(–0.925)
INTEN		–0.47***	–0.42***
		(–4.716)	(–4.163)
ROA		2.36***	1.83*
		(2.815)	(1.927)
FIXED_ASSET		–0.09	–0.17
		(–0.277)	(–0.520)
GROWTH		–0.04*	–0.03
		(–1.957)	(–1.498)
CASH_FLOW		1.40*	1.12
		(1.808)	(1.442)
RE_EARNING			0.43
			(1.068)
RET			–0.05
			(–0.623)
VOLATILITY			–0.38
			(–0.799)
OWNCON			0.62*
			(1.829)
EXCHH			0.11
			(0.794)
INDEP			–1.25
			(–1.569)
MEETING			–0.22***
			(–2.594)
SOE			0.08
			(0.882)
Constant	1.32***	–5.10***	–4.55***
	(29.053)	(–5.919)	(–4.816)
Industry FE	YES	YES	YES
Year FE	YES	YES	YES
R2	0.336	0.375	0.377
F	3.433	14.93	8.605
N	4,713	4,713	4,713

*, **, and *** denote significance at the 10, 5, and 1% levels, respectively. Standard errors are corrected for heteroskedasticity and clustering at the CEO-firm level (t-statistics are in parentheses).

From the perspective of other control variables at the firm level, the first is company size. In the second and third columns, the correlation coefficient between company size and innovation productivity is 0.32 and is significantly positive at the 1% level. This indicates that the larger the asset size of listed companies is, the more obvious the competitive advantage and the higher the innovation productivity, which indicates that the company can use the scale effect to effectively improve its innovation output efficiency. There is a significant negative correlation between the company’s capital intensity and the productivity of enterprise innovation, which indicates that the productivity of innovation is relatively low among enterprises with larger fixed costs. The negative correlation between the size of the board and innovation productivity indicates that as board size increases, decision-making efficiency decreases, thus affecting innovation productivity. In general, the regression results for the control variables and the explained variables in this paper are generally consistent with the existing literature.

#### CEO Overconfidence, Ownership Differences, and the Productivity of Firm Innovation

Furthermore, we examine the differences in the impact of CEO overconfidence on the productivity of corporate innovation among firms with different ownership types. Table 5 reports the regression results for CEO overconfidence and the innovation productivity of firms with different ownership types. The results show that in Chinese SOEs, the correlation coefficient between CEO overconfidence and enterprise innovation productivity is 0.14, which is positive but not significant; however, in private enterprises, the correlation coefficient between CEO overconfidence and enterprise innovation productivity is 0.21 and is significantly positive at the 1% level. Moreover, the difference between the coefficients on OVER_CONFIDENCE of the two subgroups is significant. The comparative analysis shows that the characteristics of CEO overconfidence may be more likely to affect the business decision-making of private enterprises, while the operation decision-making mechanism of Chinese SOEs is relatively rigid and the decision-making chain is longer. Therefore, the effect of CEO overconfidence on the company’s operation and management decision-making are significantly weakened. This finding is consistent with the expectations in this paper and supports H2.

**TABLE 5:** CEO overconfidence, ownership differences, and the productivity of firm innovation.

VARIABLES	(1)	(2)
	SOEs	Private enterprises
	LN_CITATIONS t + 1	LN_CITATIONS t + 1
OVER_CONFIDENCE	0.14	0.21***
	(1.391)	(2.732)
SIZE	0.29***	0.33***
	(4.520)	(4.831)
LEV	–0.22	–0.33
	(–0.357)	(–0.799)
INTEN	–0.41***	–0.37***
	(–2.685)	(–2.868)
ROA	3.12*	1.59
	(1.910)	(1.372)
FIXED_ASSET	–0.32	–0.11
	(–0.679)	(–0.249)
GROWTH	–0.03	–0.03
	(–0.817)	(–1.270)
CASH_FLOW	0.61	1.31
	(0.508)	(1.337)
RE_EARNING	0.27	0.35
	(0.428)	(0.638)
RET	0.05	–0.06
	(0.369)	(–0.588)
VOLATILITY	–1.97***	0.45
	(–2.677)	(0.787)
OWNCON	0.57	0.57
	(0.998)	(1.311)
EXCHH	–0.80*	0.24
	(–1.794)	(1.616)
INDEP	–1.11	–1.56
	(–0.891)	(–1.562)
MEETING	–0.09	–0.34***
	(–0.653)	(–3.329)
Constant	–4.95***	–4.02***
	(–3.952)	(–2.621)
Year FE	YES	YES
Industry FE	YES	YES
r2_a	0.444	0.351
N	1,766	2,947
F:Chow-test	4.1022***	

* and *** denote significance at the 10 and 1% levels, respectively. Standard errors are corrected for heteroskedasticity and clustering at the CEO-firm level (t-statistics are in parentheses).

### Additional Analyses

#### The Relationship Between CEOs Overconfidence and Innovation Quality Level

The more citations a patent has—in other words, the higher its citation ranking—the higher the quality of the patent, the greater its potential economic value, and therefore the higher the quality of the firm’s innovation. From the perspective of citation rate distribution, the patents applied for by listed companies are divided into patents with citation rates in the top 1%, those in the 2–10%, those below the top 10% but at least one citation, and patents with no citations. The higher the citation rate is, the higher the innovation quality. Table 6 reports the results of multiple regressions. The results show that for CEO overconfidence and LN_CITATIONS top 2–10% and LN_CITATIONS uncited, the correlation coefficients are 0.07 and 0.15, respectively, which are significantly positive at the level of 1%; however, the correlations between CEO overconfidence and LN_CITATIONS top1% and LN_CITATIONS top10%-cited are positive but not significant. The regression results of the comparative analysis show that CEO overconfidence can significantly promote the output of patents with medium to high citation rates, which is an advantage of CEO overconfidence. However, this feature also has disadvantages, i.e., the feature of overconfidence makes it difficult for CEOs to listen to the reasonable opinions of others in decision-making, resulting in the output of some patents without citations. Meanwhile, it is difficult for enterprises with overconfident CEOs to obtain patents with the highest citation rate in the market.

**TABLE 6:** CEO overconfidence and innovation quality level: Patent citation rate.

VARIABLES	(1)	(2)	(3)	(4)
	LN_CITATIONS top 1% t + 1	LN_CITATIONS top 2–10% t + 1	LN_CITATIONS top 10%-cited t + 1	LN_CITATIONS uncited t + 1
OVER_CONFIDENCE	0.02	0.07***	0.01	0.15***
	(1.615)	(3.669)	(1.423)	(2.602)
SIZE	0.07***	0.10***	0.04***	0.32***
	(5.612)	(6.307)	(4.815)	(7.729)
LEV	–0.08	–0.18*	–0.08*	–0.29
	(–1.039)	(–1.799)	(–1.647)	(–0.925)
INTEN	–0.06***	–0.12***	–0.05***	–0.45***
	(–3.338)	(–4.237)	(–2.993)	(–4.765)
ROA	0.29	0.12	0.06	2.25***
	(1.398)	(0.409)	(0.374)	(2.665)
FIXED_ASSET	–0.03	–0.14	–0.02	0.38
	(–0.442)	(–1.348)	(–0.426)	(1.232)
GROWTH	–0.00	0.00	0.00	0.01
	(–0.375)	(0.071)	(0.707)	(0.534)
CASH_FLOW	0.15	0.42*	–0.11	2.15***
	(1.009)	(1.744)	(–0.872)	(3.528)
RE_EARNING	0.01	0.06	0.06*	0.55
	(0.099)	(0.588)	(1.718)	(1.457)
RET	0.04**	0.08***	0.00	0.07
	(2.003)	(3.001)	(0.164)	(1.134)
VOLATILITY	–0.06	–0.09	0.10*	–0.43
	(–1.075)	(–0.975)	(1.832)	(–1.127)
OWNCON	0.04	0.05	–0.01	–0.07
	(0.640)	(0.536)	(–0.102)	(–0.224)
EXCHH	0.01	0.03	0.02	0.25*
	(0.302)	(0.743)	(1.039)	(1.859)
INDEP	0.08	–0.09	0.11	–1.40*
	(0.393)	(–0.323)	(0.866)	(–1.771)
MEETING	–0.03*	–0.06**	–0.01	–0.16**
	(–1.776)	(–2.336)	(–1.017)	(–1.986)
SOE	0.03	0.02	–0.01	0.04
	(1.606)	(0.821)	(–0.533)	(0.489)
Constant	–1.43***	–1.82***	–0.69***	–4.41***
	(–5.229)	(–5.207)	(–4.314)	(–4.877)
Industry FE	YES	YES	YES	YES
Year	YES	YES	YES	YES
R2	0.215	0.282	0.186	0.407
F	4.162	5.671	3.028	10.61
N	4,713	4,713	4,713	4,713

*, **, and *** denote significance at the 10, 5, and 1% levels, respectively. Standard errors are corrected for heteroskedasticity and clustering at the CEO-firm level (t-statistics are in parentheses).

#### CEO Overconfidence and Firm Innovation Direction

In addition, enterprises choose the direction of R&D innovation. It is an important channel for enterprises to break through the current development bottleneck, gain stronger bargaining power and broader market space to achieve breakthrough innovation in new fields and acquire new technology. Therefore, the choice of innovation direction will also have an important impact on enterprise value. Table 7 reports the regression results for CEO overconfidence and the direction of corporate innovation. The results show that the correlation coefficient of CEO overconfidence and LN_KNOWNCLASS is 0.09, which is significant at the 5% level. However, the correlation coefficient between CEO overconfidence and LN_KNOWNCLASS is 0.03. This correlation thus is positive but not significant, which indicates that if the CEO of an enterprise has characteristics of overconfidence, the enterprise is more likely to achieve R&D success in areas with higher innovation and breakthroughs; there is no obvious positive effect on innovation in familiar areas.

**TABLE 7:** CEO overconfidence and the direction of corporate innovation.

VARIABLES	(1)	(2)
	LN_NEWCLASS t + 1	LN_KNOWNCLASS t + 1
OVER_CONFIDENCE	0.09**	0.03
	(2.495)	(1.267)
SIZE	0.18***	0.10***
	(6.472)	(6.282)
LEV	–0.24	–0.03
	(–1.190)	(–0.277)
INTEN	–0.27***	–0.12***
	(–4.136)	(–3.488)
ROA	–0.31	0.76**
	(–0.615)	(2.444)
FIXED_ASSET	0.35*	0.31**
	(1.883)	(2.546)
GROWTH	–0.01	0.02*
	(–0.438)	(1.816)
CASH_FLOW	0.38	0.69***
	(0.940)	(2.689)
RE_EARNING	0.21	0.12
	(0.977)	(0.999)
RET	–0.02	0.01
	(–0.525)	(0.479)
VOLATILITY	–0.21	–0.26
	(–1.251)	(–1.127)
OWNCON	–0.32	–0.10
	(–1.395)	(–0.780)
EXCHH	0.04	0.06
	(0.490)	(1.246)
INDEP	–1.23***	–0.45
	(–2.612)	(–1.519)
MEETING	–0.17***	–0.04
	(–2.970)	(–1.245)
SOE	–0.04	0.04
	(–0.803)	(1.278)
Constant	–1.26**	–1.37***
	(–2.206)	(–3.983)
Industry FE	YES	YES
Year FE	YES	YES
R2	0.497	0.243
F	7.539	7.762
N	4,713	4,713

*, **, and *** denote significance at the 10, 5, and 1% levels, respectively. Standard errors are corrected for heteroskedasticity and clustering at the CEO-firm level (t-statistics are in parentheses).

#### CEO Overconfidence, Internal Control Level, and Firm Innovation Productivity

As the CEO is the core of the top management team, the CEO’s personal qualities have a significant impact on enterprise innovation; However, the impact of these qualities on the enterprise is also significantly affected by various internal management processes and control measures (Chan et al., 2020). In China, corporate governance quality is considered to be imperfect, especially internal control quality. Therefore, the Chinese Ministry of Finance has long been committed to strengthening the construction of internal controls to regulate the behavior of the CEO in the enterprise. However, internal control is a double-edged sword. Lacking of internal control may lead to CEOs pursuing personal interests, but excessive internal control will also restrict the decision-making power of CEOs, which is also not conducive to the development of the enterprise. Then, for enterprises with different levels of internal control, this paper conducted a test and analysis of whether the impact of CEOs on enterprise innovation exhibits differentiation.

Furthermore, we examine the impact of CEO overconfidence on the productivity of corporate innovation under different levels of internal control. We calculate firms’ internal control level following Li et al. (2019) and Chan et al. (2020) and divide the results into 4 subgroups according to the level of internal control, namely, below 25%, 25–50%, 50–75%, and above 75%; the larger the figure is, the stronger the firm’s internal control. Table 8 reports the regression results for the relationship between CEO overconfidence and the innovation productivity of listed companies under different levels of internal control. The results show that among the enterprises with an internal control level between 25 and 75%, CEO overconfidence can significantly improve the productivity of innovation. When the internal control is too low or too high, namely, the internal control level is below 25% or above 75%, respectively, the relationship between CEO overconfidence and firm innovation productivity is not significant. The results show that a high level of internal control might constrain CEOs’ decision-making power since decision-making processes in such firms are highly standardized and lack flexibility. Especially in innovation activities with high investment and high risk, it is difficult for the CEO to play a subjective role and significantly affect the innovation activities of the enterprise through personal will. Interestingly, if the internal control level of a firm is too low, it may lead to an increase in agency costs, thus hindering the efficiency and productivity of innovation.

**TABLE 8:** CEO overconfidence, internal control level, and firm innovation productivity.

VARIABLES	(1)	(2)	(3)	(4)
	Very strong (75–100%)	Strong (50–75%)	Medium (25–50%)	Weak (25–0%)
	ln_citations	ln_citations	ln_citations	ln_citations
OVER_CONFIDENCE	0.06	0.19*	0.26**	0.14
	(0.606)	(1.903)	(2.282)	(1.121)
SIZE	0.30***	0.26***	0.24***	0.45***
	(5.019)	(3.494)	(3.113)	(4.835)
LEV	–0.06	–0.16	–0.26	–0.77
	(–0.132)	(–0.295)	(–0.401)	(–1.191)
INTEN	–0.61***	–0.29**	–0.32*	–0.48**
	(–4.279)	(–1.977)	(–1.697)	(–2.521)
ROA	3.89**	0.77	1.04	3.39
	(2.480)	(0.581)	(0.646)	(1.549)
FIXED_ASSET	0.23	0.37	–0.21	–0.69
	(0.480)	(0.665)	(–0.328)	(–1.195)
GROWTH	–0.01	–0.06	–0.09**	0.05
	(–0.352)	(–1.372)	(–1.984)	(0.853)
CASH_FLOW	–0.65	0.61	–0.20	3.38**
	(–0.429)	(0.379)	(–0.138)	(2.387)
RE_EARNING	0.29	0.78	0.81	–0.53
	(0.582)	(1.285)	(1.067)	(–0.579)
RET	–0.07	0.03	0.08	–0.14
	(–0.427)	(0.183)	(0.493)	(–0.785)
VOLATILITY	0.07	–0.43	–1.86*	–1.61
	(0.090)	(–0.795)	(–1.676)	(–1.028)
OWNCON	0.30	0.26	0.80	0.64
	(0.575)	(0.478)	(1.258)	(0.927)
EXCHH	0.08	0.29	–0.32	0.50
	(0.360)	(1.368)	(–1.348)	(1.583)
INDEP	–0.55	–1.10	–0.22	–2.87*
	(–0.482)	(–0.818)	(–0.140)	(–1.784)
MEETING	–0.32**	–0.14	–0.04	–0.40**
	(–2.481)	(–0.972)	(–0.235)	(–2.117)
SOE	0.08	0.11	0.01	0.10
	(0.658)	(0.785)	(0.092)	(0.674)
Constant	–2.83**	–4.03**	–2.66	–5.84**
	(–2.152)	(–2.516)	(–1.635)	(–2.559)
Year FE	YES	YES	YES	YES
Industry FE	YES	YES	YES	YES
r2_a	0.410	0.357	0.344	0.376
F	4.78	2.76	2.27	4.41
N	1,272	1,300	1,142	999
F:Chow-test	3.8202***		2.7656***	

*, **, and *** denote significance at the 10, 5, and 1% levels, respectively. Standard errors are corrected for heteroskedasticity and clustering at the CEO-firm level (t-statistics are in parentheses).

Moreover, the results of Chow test show that the difference between the coefficients on OVER_CONFIDENCE of the two subgroups (below 25% vs. 25 to 50%) is significant. In addition, the results of Chow test show that the difference between the coefficients on OVER_CONFIDENCE of the two subgroups (50–75%, and above 75%) is significant, which further supports our findings.

## Robustness Tests

### Replacing the Measure of CEO Overconfidence

In the main test of this article, the degree of CEO overconfidence is measured through the comprehensive scoring of five indicators of CEO overconfidence. To test the robustness of the main results in this paper, this paper selects four indicators out of these five, uses two combination methods for the 4 indicators, rerates CEO overconfidence and further tests the impact of CEO overconfidence on the productivity of enterprise innovation. The first method is to adopt the combination of the gender indicator, formal education level indicator, professional business education background indicator and position duality indicator, and the second method is to adopt the combination of the age indicator, gender indicator, formal education level indicator and professional business education background indicator. We take the average of the sum of the four indicators. OVER_CONFIDENCE equals 1 if the sum of the four indicators of the sample is 3 or 4; otherwise, OVER_CONFIDENCE is 0. Table 9 reports the relationship between CEO overconfidence and the productivity of innovation after the change in the measurement method. The correlation coefficients of CEO overconfidence and the productivity of enterprise innovation using the above measurement methods are 0.68 and 0.6, respectively, indicating significant and positive correlation at the level of 1%, proving the robustness of the baseline regression results of this paper.

**TABLE 9:** Robustness test: Replacing the measure of CEO overconfidence.

VARIABLES	(1)	(2)
	LN_CITATIONS t + 1	LN_CITATIONS t + 1
OVER_CONFIDENCE	0.68***	0.60***
	(3.276)	(3.267)
SIZE	0.31***	0.31***
	(7.037)	(7.082)
LEV	–0.31	–0.29
	(–0.904)	(–0.854)
INTEN	–0.42***	–0.42***
	(–4.170)	(–4.233)
ROA	1.88**	1.81*
	(1.978)	(1.896)
FIXED_ASSET	–0.13	–0.17
	(–0.392)	(–0.499)
GROWTH	–0.03	–0.03
	(–1.504)	(–1.415)
CASH_FLOW	1.12	1.09
	(1.441)	(1.413)
RE_EARNING	0.43	0.43
	(1.068)	(1.049)
RET	–0.05	–0.04
	(–0.603)	(–0.559)
VOLATILITY	–0.41	–0.41
	(–0.887)	(–0.872)
OWNCON	0.61*	0.68**
	(1.789)	(1.995)
EXCHH	0.06	0.06
	(0.398)	(0.451)
INDEP	–1.39*	–1.33*
	(–1.738)	(–1.662)
MEETING	–0.21**	–0.21**
	(–2.570)	(–2.552)
SOE	0.08	0.06
	(0.921)	(0.732)
Constant	–4.77***	–4.81***
	(–5.033)	(–5.073)
Industry FE Year FE	YES	YES
	YES	YES
R2	0.378	0.378
F	8.801	8.667
N	4,713	4,713

*, **, and *** denote significance at the 10, 5, and 1% levels, respectively. Standard errors are corrected for heteroskedasticity and clustering at the CEO-firm level (t-statistics are in parentheses).

### Additional Lagged Effects of the Independent Variables

The independent variable is lagged for two and three periods. In fact, it will take a period of time for the CEO to have a substantial impact on enterprise R&D innovation and promote the improvement of innovation productivity. Hence, in line with Choi et al. (2011), this paper applies two to three lags to the dependent variables and control variables to test the robustness of the previous main regression results. Table 10 presents the results. It shows that there is a significant positive correlation between CEO overconfidence and the productivity of corporate innovation, which supports the robustness of the results in this paper.

**TABLE 10:** Robustness test: Lagged effects of independent variables.

VARIABLES	(1)	(2)
	LN_CITATIONS t + 2	LN_CITATIONS t + 3
OVER_CONFIDENCE	0.24***	0.22***
	(3.320)	(2.986)
SIZE	0.35***	0.30***
	(5.703)	(5.074)
LEV	–0.05	0.12
	(–0.115)	(0.276)
INTEN	–0.40***	–0.36**
	(–2.701)	(–2.557)
ROA	1.75	0.82
	(1.069)	(0.488)
FIXED_ASSET	–0.36	–0.17
	(–0.793)	(–0.378)
GROWTH	–0.01	–0.01
	(–0.211)	(–0.143)
CASH_FLOW	–0.15	1.26
	(–0.119)	(1.019)
RE_EARNING	1.14*	1.33**
	(1.656)	(2.034)
RET	0.22*	0.36***
	(1.925)	(2.622)
VOLATILITY	–0.13	–0.77
	(–0.132)	(–0.674)
OWNCON	0.77	0.90*
	(1.596)	(1.883)
EXCHH	–0.04	–0.01
	(–0.214)	(–0.033)
INDEP	–1.29	–1.46
	(–1.160)	(–1.356)
MEETING	–0.35***	–0.32**
	(–3.094)	(–2.546)
SOE	0.11	0.08
	(0.980)	(0.754)
Constant	–5.47***	–4.74***
	(–4.151)	(–3.733)
Industry FE	YES	YES
Year FE	YES	YES
R2	0.379	0.357
F	5.276	5.056
N	2,555	1,936

*, **, and *** denote significance at the 10, 5, and 1% levels, respectively. Standard errors are corrected for heteroskedasticity and clustering at the CEO-firm level (t-statistics are in parentheses).

### Change the Regression Model

To further verify the robustness of the results of this paper, on the one hand, this paper further controls for the fixed effect of the industry-year in the model to prevent the estimation error caused by the omission of variables; on the other hand, as the explained variables used in this paper are all greater than 0, they are typical left-censored data. To ensure the effectiveness of the estimation coefficient, this paper uses a tobit model for machine matching. The following table reports the regression results after the change in the regression model; the first column shows the regression results after adding the fixed effect of the industry-year cross product term, and the second column shows the regression results of the tobit model. As listed in Table 11, the relationship between CEO overconfidence and the productivity of corporate innovation is significantly positive regardless of whether the fixed effect of annual traffic volume in the industry is controlled or the tobit model is adopted. The 2 methods further demonstrate the robustness of the results in our current study.

**TABLE 11:** Robustness test: Change in the regression model.

VARIABLES	(1)	(2)
	LN_CITATIONS t + 1	LN_CITATIONS t + 1
OVER_CONFIDENCE	0.16***	0.38***
	(2.613)	(3.408)
SIZE	0.29***	0.62***
	(6.468)	(9.530)
LEV	–0.21	–1.19*
	(–0.611)	(–1.915)
INTEN	–0.42***	–1.15***
	(–4.101)	(–5.784)
ROA	1.47	1.35
	(1.529)	(0.780)
FIXED_ASSET	–0.16	–0.05
	(–0.464)	(–0.082)
GROWTH	–0.00	–0.04
	(–0.024)	(–0.791)
CASH_FLOW	2.13***	3.68**
	(2.640)	(1.970)
RE_EARNING	0.53	1.50**
	(1.351)	(2.128)
RET	0.08	–0.02
	(0.880)	(–0.104)
VOLATILITY	–0.17	–1.28
	(–0.320)	(–1.189)
OWNCON	0.40	1.51**
	(1.142)	(2.438)
EXCHH	0.12	0.23
	(0.846)	(0.964)
INDEP	–1.13	–3.58***
	(–1.410)	(–2.599)
MEETING	–0.22***	–0.59***
	(–2.585)	(–3.288)
SOE	0.08	0.16
	(0.966)	(1.231)
Constant	–4.07***	–10.29***
	(–4.246)	(–6.102)
Industry FE	YES	YES
Year FE	YES	YES
Industry_year FE	YES	
R2	0.421	.
F	7.409	.
N	4,643	4,715

*, **, and *** denote significance at the 10, 5, and 1% levels, respectively. Standard errors are corrected for heteroskedasticity and clustering at the CEO-firm level (t-statistics are in parentheses).

### Propensity Score Matching Analysis

The endogenous choice of overconfident CEOs could also affect our analysis. For example, companies with a strong willingness to innovate may be more likely to hire an overconfident CEO than other companies. To address this concern, we adopt a propensity score matching approach. For each firm with an overconfident CEO (a CEO who scored a 4 or 5 in our analysis of five overconfidence indicators), we identify a matched control firm with a less overconfident CEO (below the median level) and calculate the average difference in innovation productivity for all matched pairs. To find the matched firms, we employ radius matching and nearest neighbor matching methods (Guo and Fraser, 2010). Our matching covariates include all control variables in our baseline regression. In addition, we ensure that the matched firm is in the same fiscal year and industry as the treated firm, which allows us to compare companies that are similar in observable characteristics but differ in CEO overconfidence levels.

As reported in columns 1 and 2 of Table 12, the average treatment effects are positively significant for both the radius matching and nearest neighbor matching methods. Our matching results show that a firm with an overconfident CEO is, on average, more innovative than its counterpart with a less overconfident CEO.

**TABLE 12:** Propensity score matching analysis.

VARIABLES	(1)	(2)
	Nearest neighbor matching	Radius matching
	LN_CITATIONS t + 1	LN_CITATIONS t + 1
OVER_CONFIDENCE	0.17***	0.17***
	(2.779)	(2.774)
SIZE	0.32***	0.32***
	(7.123)	(7.144)
LEV	–0.31	–0.32
	(–0.909)	(–0.934)
INTEN	–0.42***	–0.41***
	(–4.131)	(–4.107)
ROA	1.91**	1.89**
	(1.994)	(1.970)
FIXED_ASSET	–0.19	–0.15
	(–0.565)	(–0.449)
GROWTH	–0.03	–0.04
	(–1.580)	(–1.640)
CASH_FLOW	1.13	1.15
	(1.457)	(1.474)
RE_EARNING	0.41	0.39
	(1.009)	(0.960)
RET	–0.05	–0.04
	(–0.677)	(–0.549)
VOLATILITY	–0.37	–0.39
	(–0.780)	(–0.818)
OWNCON	0.60*	0.61*
	(1.747)	(1.796)
EXCHH	0.10	0.10
	(0.745)	(0.716)
INDEP	–1.22	–1.28
	(–1.531)	(–1.613)
MEETING	–0.22***	–0.22***
	(–2.620)	(–2.643)
SOE	0.07	0.07
	(0.864)	(0.861)
Constant	–4.53***	–4.55***
	(–4.786)	(–4.789)
Year FE	YES	YES
Industry FE	YES	YES
r2_a	0.378	0.379
F	8.574	8.542
N	4,678	4,661

*, **, and *** denote significance at the 10, 5, and 1% levels, respectively. Standard errors are corrected for heteroskedasticity and clustering at the CEO-firm level (t-statistics are in parentheses).

## Conclusion

Drawing on a sample of public firms listed on the Shanghai and Shenzhen stock exchanges in China between 2009 and 2016, the current study examines how CEO overconfidence could influence the productivity, quality and direction of corporate innovation. We find that CEO overconfidence is positively related to a firm’s innovation productivity, as measured by the number of granted patents with citations. This relation is statistically significant and consistent with those of studies on the Western context. This finding is robust after accounting for endogeneity. In addition, many Chinese firms are state-owned and controlled by SASAC (Bai et al., 2006; Bruton et al., 2015). Using data from SOEs and non-SOEs in China, this study provides novel evidence that for Chinese SOEs, the correlation coefficient between CEO overconfidence and enterprise innovation productivity is not significant. However, in private enterprises, the correlation coefficient between CEO overconfidence and enterprise innovation productivity is positive and significant at the 1% level. This result could be explained by the fact that CEOs of Chinese state-owned firms are less influential in the firm’s decision-making process; therefore, his/her personal characteristics might be less influential than those of a CEO of a Chinese private firm.

Furthermore, our results show that CEO overconfidence can significantly promote the output of patents with medium to relatively high citation rates (patents in the top 2–10% of citation rates), which is an advantage of CEO overconfidence. However, it is difficult for enterprises with overconfident CEOs to obtain patents with the highest quotation rate (top 1% citations) in the market. In addition, we find that if the CEO of an enterprise has the characteristics of overconfidence, the enterprise is more likely to achieve R&D success in new areas, while the promotion effect of overconfident CEOs on the enterprise’s R&D success in familiar knowledge areas is not obvious.

In addition, we examine the impact of CEO overconfidence on innovation productivity under different levels of internal control. Our results show that among enterprises with an internal control level between 25 and 75%, CEO overconfidence can significantly improve the productivity of innovation. When internal control is too high or internal control is too low, the relationship between CEO overconfidence and firm innovation productivity is not significant. The results show that strong internal control might constrain CEOs’ decision-making power since the firm’s decision-making process is highly standardized and lacks flexibility. However, if the internal control of a firm is too low, it may lead to an increase in agency costs, thereby hindering the efficiency and productivity of innovation.

We acknowledge that our study has several limitations. First, we use only patent-based measures as proxies for innovation productivity, innovation quality and innovation direction. Second, since our sample covers relatively large Chinese firms, it is not clear whether these results are generalizable to small firms in China. Third, we acknowledge that the measure of CEO overconfidence used in this study is indirect and imperfect. Therefore, we suggest that future research contribute to the literature by exploring new measures of innovation and CEO overconfidence. In addition, considering the availability of data, qualitative studies, such case studies, on the effects of CEO overconfidence on firm innovation based on small Chinese firms could be conducted to determine the specific mechanisms of these effects.

## Data Availability Statement

Publicly available datasets were analyzed in this study. This data can be found here: Anyone who wants to obtain the data should contact CSMAR directly since the original data is commercially available by CSMAR.

## Author Contributions

YZ contributed to the writing and formatting of the research. ZL contributed to the editing and analysis of the research. Both authors contributed to the article and approved the submitted version.

## Conflict of Interest

The authors declare that the research was conducted in the absence of any commercial or financial relationships that could be construed as a potential conflict of interest.

## Publisher’s Note

All claims expressed in this article are solely those of the authors and do not necessarily represent those of their affiliated organizations, or those of the publisher, the editors and the reviewers. Any product that may be evaluated in this article, or claim that may be made by its manufacturer, is not guaranteed or endorsed by the publisher.
